# COVID-19 pandemic: guidance for nuclear medicine departments

**DOI:** 10.1007/s00259-020-04825-8

**Published:** 2020-04-15

**Authors:** D. Paez, G. Gnanasegaran, S. Fanti, J. Bomanji, M. Hacker, M. Sathekge, H. S. Bom, J. J. Cerci, A. Chiti, K. Herrmann, A. M. Scott, J. Czernin, N. El-Haj, E. Estrada, O. Pellet, P. Orellana, F. Giammarile, M. Abdel-Wahab

**Affiliations:** 1 Division of Human Health, International Atomic Energy Agency, PO Box 100, 1400 Vienna, Austria; 2 Royal Free Hospital, London, UK; 3 Department of Oncology, Division of Nuclear Medicine, University of Bologna, Bologna, Italy; 4 Institute of Nuclear Medicine, University College London Hospital, London, UK; 5 Division of Nuclear Medicine, Department of Nuclear Medicine Biomedical Imaging and Image-guided Therapy, Medical University of Vienna, Vienna General Hospital, Vienna, Austria; 6 Nuclear Medicine Department, University of Pretoria and Steve Biko Academic Hospital, Pretoria, South Africa; 7 Department of Nuclear Medicine, Chonnam National University Medical School, Hwasun, South Korea; 8 PET/CT Department at Quanta Diagnostics and Therapy, Curitiba, Brazil; 9 Humanitas University and Humanitas Research Hospital, Milan, Italy; 10 Department of Nuclear Medicine, Universitätsklinikum Essen, Essen, Germany; 11 Department of Molecular Imaging and Therapy, Austin Health, Melbourne, Australia; 12 Ahmanson Translation Imaging Division, David Geffen School of Medicine at UCLA, Los Angeles, CA USA

## Background

Coronaviruses are non-segmented, enveloped positive-sense ribonucleic acid viruses from the Coronaviridae family. There are six types of the coronavirus known to infect humans. Four of them cause mild respiratory symptoms, while two of them, the Middle East respiratory syndrome coronavirus (MERS) and the severe acute respiratory syndrome (SARS), have caused epidemics with high mortality rates [1, 2].

In December 2019, a new type of coronavirus 2019-nCoV/SARS-CoV-2, causing COVID-19 disease, was extracted and identified from the lower respiratory tract samples of several patients in Wuhan, China [3]. These patients presented with symptoms of severe pneumonia, including fever, fatigue, dry cough, and respiratory distress.

The coronavirus disease 19 (COVID-19) is a highly transmittable and pathogenic viral infection. It is believed to be transmitted via respiratory droplets and fomites during close unprotected contact between an infector and an infectee. The coronaviruses mainly infect epithelial cells in the lung, but SARS-CoV-2 has been detected in respiratory, fecal, and blood specimens of patients infected with the virus [4].

On February 3, 2020, the World Health Organization declared a public health emergency of international concern, and on March 11, declared COVID-19 a pandemic [5]. The total number of confirmed cases, deaths associated with COVID-19, and affected countries and territories continues to grow; detailed statistics can be found at the WHO–Coronavirus disease (COVID-19) Pandemic site [5] or the John Hopkins Coronavirus Resource Centre. [6].

Health care providers around the world are facing challenging decisions. They are rapidly adjusting their standard operating procedures (SOPs) to cope with the pandemic cases and deliver their services. This is done in line with local guidance, resources available, and the advice of the World Health Organization (WHO) Minimum Requirements for infection prevention and control (IPC) programmes [7].

This publication was prepared based on the systematic review of available literature on the subject and the contribution of a panel of international experts during the webinar entitled “Coronavirus disease (COVID-19) Pandemic: Challenges for the Nuclear Medicine Departments,” organized by the International Atomic Energy Agency (IAEA) and broadcasted live on Wednesday 25 March 2020 [8].

The objective of this guide is not to override any local guidance or national practice guidelines or rules, nor does it provide comprehensive advice on all aspects of nuclear medicine practice. It is solely intended as advice for nuclear medicine facilities during this time of adjustment and adaptation to the COVID-19 pandemic. We present suggested recommendations for nuclear medicine departments to follow, based on a typical patient’s “journey” through the department.

## General measures

During the COVID-19 pandemic, SOPs in nuclear medicine (NM) departments should be adapted. In addition to the established procedures, special emphasis must be placed on minimizing the risk to staff, patients, and family members, as well as controlling the transmission of the virus while continuing to provide the essential and critical services.

The World Health Organization (WHO) has published the “COVID-19: Operational Guidance for maintaining essential health services during an outbreak” [9] which includes six main processes that can be extrapolated to nuclear medicine facilities (Table 1).

**Table 1:** Operational Processes. Adapted from “WHO–COVID-19: Operational Guidance for maintaining essential health services during an outbreak”

I. Establish simplified purpose-designed governance and coordination mechanisms	
II. Identify context-relevant essential services	
III. Optimize service delivery settings and platforms	
IV. Establish effective patient flow (screening, triage, and targeted referral) at all levels	
V. Rapid re-distribution of health workforce capacity, including re-assignment of tasks	
VI. Identify mechanisms to maintain the availability of essential equipment and supplies	

## Establish simplified purpose-designed governance and coordination mechanisms

NM departments must be flexible and adapt, considering the stage of the epidemic in the population they serve.

- Establish a COVID-19 Incident Management Team and designate a focal point.
- Consideration for the reallocation of human, financial and material resources, and mobilizing additional resources are essential.
- All staff members should receive specific training in identifying COVID-19 symptoms, hygiene procedures, handling COVID-19 patients, disinfection procedures, and use of personal protective equipment (PPE), among others [10, 11].
- Managers must inform staff not to report to work if they feel unwell or have any suspicion of having COVID-19 symptoms.
- Facilities must ensure that the waiting area has access to handwashing facilities and that hand sanitizers, tissue boxes, and masks are within reach so that patients can follow basic hygiene practices. The waiting areas need to be organized in such a way that it provides space for patients to sit at a distance from each other to reduce the risk of transmission.
- It is essential to ensure strict hand hygiene, maintaining at least 1 m (3 ft) distance [5] in all patient/staff interactions when possible, avoiding crowding at the workplace, etc.
- Implementation of cleaning and disinfecting procedures for equipment and accessories including camera gantries, patient beds, blood pressure cuffs, workstations, mouse, keyboards, and any other items of daily use.
- Develop a contingency and business continuity plan if one of the staff becomes sick with COVID-19.

## Identify context-relevant essential services

NM departments should continue their operation. It is essential to exert flexibility and adapt the patient schedule to maintain the provision of essential services. Special attention must be paid to adjust the provision of services, taking into account the phase of the epidemic in the local population served.

Essential procedures (scans and therapies) must be identified and prioritized. Postponing non-essential procedures as well as research activities should be considered. Teaching activities should be given through dedicated teleconferencing software or postponed. It is essential to establish a work plan to reinstate delayed services or events [12–15].

## Optimize service delivery settings and platforms

- Use of communication technologies must be a primary consideration.
- Teleconsulting with patients prior to scheduling and the day before attending the nuclear medicine center must be established, in order to identify patients who may have COVID-19 symptoms.
- Patients should be instructed to attend their appointments alone, if their condition allows. In case assistance is required, only one accompanied person, ideally without risk factors, should be permitted. The risk factors should be stated during the phone appointment.
- Remote reporting should be considered, when possible, provided that national or local rules are followed.
- Teleconsultation with patients after radionuclide therapies is strongly encouraged.
- Establish virtual multidisciplinary meetings.
- Establish remote communication channels with referring physicians.

## Establish effective patient flow (screening, triage, and targeted referral) at all levels

In addition to the established procedures including radiation protection, it is crucial to guarantee the adherence to recommendations, including (1) distancing, (2) hygiene, (3) separate spaces for patients with known or suspected COVID-19 to prevent spread, and (4) ensuring supplies are available.

Departments sited in COVID-19 hot spot zones should instruct all front-line personnel to rigorously follow local PPE guidance before dealing with patients.

The possibility of airborne transmission of COVID-19 continues to be debated [5, 16]. It is known that airborne viruses can spread in air conditioning and ventilation systems and therefore it can be expected that certain medical procedures associated with the generation of aerosols, such as ventilations scans and oxygen supplementation, carry an increased risk of transmission. Therefore, alternative options for test or procedure should be considered or special precautions especially for personnel conducting these tests must be taken.

### Patient arrival, waiting area

- Reception staff to be sited behind a glass or plastic screen if not already in place. Upon arrival, patients are asked to declare if they have been in contact with patients with COVID-19 or if they have any symptoms.
- It is useful to display information announcements at the reception, indicating possible symptoms and asking patients to inform staff if they have symptoms.
- Display posters to promote handwashing and proper respiratory hygiene measures.
- Consideration should be given to implement temperature control using electronic skin contact systems.
- Be vigilant in identifying patients with symptoms of COVID-19.
- Consider that there are asymptomatic patients and apply all protective and hygiene measures.
- When patients with COVID-19 are identified, they should be placed in a separate waiting area, and if available, appropriate consultation with the infectious diseases team should be sought.
- COVID-19 patients should wear a surgical mask to minimize the risk of transmission.
- Consider providing surgical masks to all patients and chaperones, to wear at all times while in the NM center. Remember that local or national regulations must be followed.

### During the injection and scan

- Use all aseptic and antiseptic techniques
- Apply all standard radiation protection and optimisation principles;
- Use the appropriate PPE
- Place special attention when removing the gloves and other protective elements
- Disinfect the devices used during patient preparation and injection
- Thoroughly sanitize hands after each procedure
- Dispose the used protective elements in a container for biosafety waste

### During the scan

- Apply all standard radiation protection and optimisation principles
- Use the appropriate PPE
- Use disposable protective elements for the scanners

### When the patient is scanned and goes home

- After scanning a COVID-19 patient, scanners and room surfaces should be disinfected to prevent possible spread of infection. If available, local recommendations and guidelines should be followed, e.g., Public Health England has released a guide for disinfecting scanners and clinic rooms with solutions containing 1000 ppm available chlorine, and appropriate training for environmental maintenance personnel is recommended.
- Before the patient is released, if the hybrid study involves a CT of the chest, it is imperative to look for incidental COVID-19 findings that might suggest COVID-19 infection.
- If incidental COVID-19 findings are detected in the lungs, it must be reported immediately to the referring clinician, and the patient triaged for the appropriate care pathway.

## Rapid re-distribution of health workforce capacity, including re-assignment of tasks

Health care personnel are at a high risk for exposure and susceptible to contracting the virus. For this reason, we must be vigilant in recognizing symptoms [17].

- The same precautions and screening tests that apply to patients upon arrival should be implemented for the nuclear medicine staff.
- Simple measures, such as staying home if you are not feeling well, are essential to reducing the risk of infection and transmission to the team.
- Consider segregating staff into teams to reduce the possibility of virus transmission between health care providers which could result in the inability of the department to function.
- Consider re-training of staff to cover other positions within the department.
- All required personal protective equipment (PPE) should be made available for staff at all times and all working sites. It is recommended to follow guidelines of the WHO–Rational use of personal PPE for coronavirus disease (COVID-19) [10].
- Consider providing staff transportation and, if necessary, staff accommodation.
- Environmental services staff members who clean all departmental areas during, and out of, working hours must be specifically trained for professional cleaning of potentially contaminated surfaces after each contact with a high-risk patient.
- Establish periodic virtual staff meetings to update on the local status of the pandemic and enquire about their well-being.
- Psychological consultation for staff should be available.

## Identify mechanisms to maintain the availability of essential equipment and supplies

Nuclear medicine centers rely on the availability of radioisotopes that are produced in a limited number of facilities worldwide and depend on adequate distribution channels. Due to the pandemic, the availability of international flights, including cargo, has been dramatically reduced. Shortages of kits and radioisotopes are therefore expected.

At this point in time, some countries in Africa, Asia, and Latin America have reported shortages of radioiodine and have been informed by providers of the possible shortage of molybdenum-technetium generators.

It is essential to ensure, as far as possible, the uninterrupted operation of cyclotrons to ensure the provision of PET services to oncologic patients. In the likely scenario where there is a shortage of supplies for SPECT studies, it might be useful to explore additional clinical applications of PET including cardiac, neurological, and infection/inflammation studies.

It is essential for managers of nuclear medicine facilities and cyclotron centres to make a:

- List of required supplies
- List of all possible suppliers and distribution channels
- Maintain a detailed inventory and to coordinate the redistribution of supplies

## Conclusion

The current COVID-19 pandemic poses many challenges for the practice of nuclear medicine. If adequately prepared, departments can continue to deliver their essential services, while mitigating the risk for patients and staff. This requires adapting the SOPs, as quickly as possible, to meet the new requirements.

A multifactorial approach covering all elements from patient scheduling to reporting scans and paying attention to incidental COVID-19 suspected findings, must be adopted. A summary of which is presented in Table 2.

**Table 2:** Essential considerations

The robust screening process for patients and staff	
Identification of cases promptly	
Social distancing	
Training of staff members	
Posters to promote handwashing and proper respiratory hygiene	
Cleaning and disinfection of equipment and accessories	
Hand sanitizing dispensers	
Stay at home or working from home guidelines for staff	
Develop a contingency and business continuity plan	

Individual hygiene, disinfecting and cleaning, as well as the use of appropriate PPE, must be emphasized and practiced.

The intention of this guide is to provide support to nuclear medicine centers for the COVID-19 pandemic; a more detailed guide is being prepared by the IAEA and will be available in due course.
